# Diagnostic Value of 2-[^18^F]FDG PET/CT in a Patient with Atypical Subacute Thyroiditis: A Case Report

**DOI:** 10.3390/life12081217

**Published:** 2022-08-10

**Authors:** Teresa Kraus, Marcus Hacker, Werner Langsteger, Shuren Li, Raffaella Calabretta

**Affiliations:** Division of Nuclear Medicine, Department of Biomedical Imaging and Image-Guided Therapy, Medical University of Vienna, 1090 Vienna, Austria

**Keywords:** 2-[^18^F]-FDG, PET/CT, subacute thyroiditis, FUO, IUO

## Abstract

**Simple Summary:**

Positron emission tomography/computed tomography (PET/CT) imaging with 2-deoxy-2-[^18^F]fluoro-d-glucose (2-[^18^F]FDG) could be a useful diagnostic tool to detect foci of infection or inflammation in patients with fever of unknown origin (FUO) or with inflammation of unknown origin (IUO). We report a case of a patient originally presenting with a clinical history of FUO and later with persistent high-sensitivity C-reactive protein (hsCRP) levels, even after antibiotic therapy. The patient underwent 2-[^18^F]FDG PET/CT to investigate and to localize a possible focus of infection or inflammation. Since only 2-[^18^F]FDG hotspots were detected in both thyroid lobes, specific thyroid diagnostic examinations were performed. Subacute thyroiditis (SAT) was then diagnosed, and other possible causes of FUO or IUO were excluded. We found it interesting to present this case to illustrate the potential diagnostic value of 2-[^18^F]FDG PET/CT imaging in patients with atypical SAT presenting only with FUO.

**Abstract:**

Background: Positron emission tomography/computed tomography (PET/CT) imaging with 2-deoxy-2-[^18^F]fluoro-d-glucose (2-[^18^F]FDG) is a sensitive diagnostic imaging modality in oncology and could be a useful diagnostic tool in patients with fever of unknown origin (FUO) or with inflammation of unknown origin (IUO). Case presentation: We report a case of a patient originally presenting with a clinical history of FUO and later with persistent high-sensitivity C-reactive protein (hsCRP) levels, even after antibiotic therapy. The patient underwent 2-[^18^F]FDG PET/CT to investigate and to localize a possible focus of infection or inflammation. 2-[^18^F]FDG hotspots were detected in both thyroid lobes. Thyroid diagnostic examinations and follow up were performed. Subacute thyroiditis (SAT) was then diagnosed by thyroid examinations, and other possible causes of FUO or IUO were not found. Conclusion: This case illustrates the potential diagnostic value of 2-[^18^F]FDG PET/CT in patients with atypical SAT, who originally present with only a clinical history of FUO.

## 1. Introduction

Positron emission tomography/computed tomography (PET/CT) imaging with 2-deoxy-2-[^18^F]fluoro-d-glucose (2-[^18^F]FDG) is a useful diagnostic imaging procedure providing both tomographic and functional information. 2-[^18^F]FDG PET imaging has been proven to be a sensitive imaging modality in oncology and has been proposed also to detect foci of infection or inflammation in patients with fever of unknown origin (FUO) or with inflammation of unknown origin (IUO). Indeed, 2-[^18^F]FDG is actively incorporated in vivo by activated leucocytes, monocyte, macrophages, and CD4+ T-lymphocytes accumulating at the sites of infection [1,2,3,4,5]. Typical subacute thyroiditis (SAT) is characterized by a painful swelling of the thyroid gland, fever, and an initial hyperthyroidism phase, often followed by transient or permanent hypothyroidism. It often follows a viral upper respiratory tract infection. Painless SAT is less common and histologically similar to Hashimoto’s thyroiditis and drug-related destructive thyroiditis [6]. To the best of our knowledge, there have only been a few published case studies about patients with SAT, who underwent a 2-[^18^F]FDG PET [7,8,9]. We found it interesting to report the case of a patients with SAT, who underwent 2-[^18^F]FDG PET/CT because heoriginally had only a clinical history of FUO.

## 2. Detailed Case Description

Written informed consent for the publication of this case report was obtained from the patient.

### 2.1. Clinical History

A 41-year-old patient with no medical history or known thyroid disorder presented to the Department of Nuclear Medicine at University Hospital Vienna with FUO over the last couple of weeks and elevated high-sensitivity C-reactive protein (hsCRP) levels, even after antibiotic therapy, to perform a 2-[^18^F]FDG PET/CT. Moreover, he presented with hyperhidrosis and a light feeling of pressure on the neck. Previously, the patient was scheduled by the Department of Cardiothoracic Surgery at University Hospital Vienna for an aortic valve congenital heart defect with profound aortic valve insufficiency and aortic valve stenosis, bicuspid applied aortic valve. There, the patient was admitted for a planned surgical rehabilitation of the combined aortic valve congenital heart defect. Initial laboratory tests on admission to the hospital showed a normal white cell count, hemoglobin level, and platelet count. hsCRP level was mildly elevated at 2.01 mg/L. A standard thyroid function test was also performed revealing regular thyroid function with normal free triiodothyronine (fT3), free thyroxine (fT4), and thyroid-stimulating hormone (TSH) levels. A chest X-ray performed on the day of admission reported no pulmonary consolidation or pleural effusion. The patient reported headache. Because of the patient’s laboratory results and the clinical presentation, the planned surgery was not carried out. The patient was sent home with a prescribed antibiotic therapy for suspected acute sinusitis. Treatment was started with amoxicillin 1000 mg once a day for a week, followed by amoxicillin/clavulanic acid 875 mg/125 mg twice a day also for a week, without any significant clinical improvement and persistent elevated hsCRP levels.

### 2.2. Screening Tests and Diagnosis

**Figure 1:**
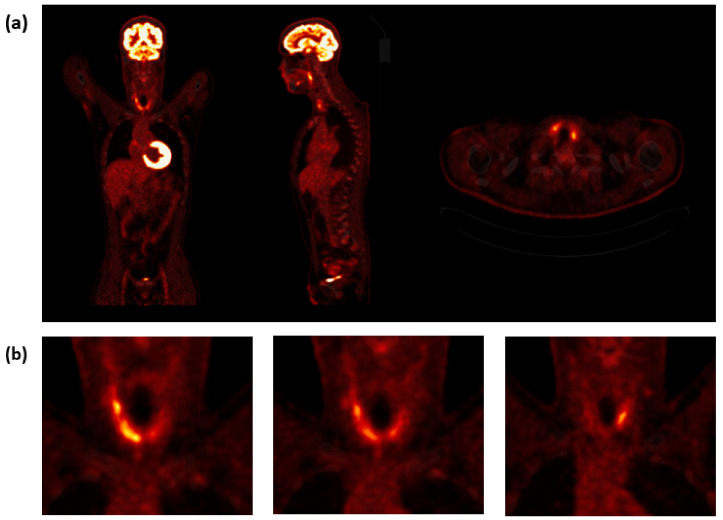
**2-[^18^F]FDG PET/CT imaging.** (**a**) Coronal, sagittal, and transversal 2-[^18^F]FDG PET/CT images slices showing high tracer uptake in both lobes of the thyroid. (**b**) Representative coronal PET/CT images of multiple 2-[^18^F]FDG-hot spots in both thyroid lobes, mostly on the right. PET/CT, positron emission tomography/computed tomography; 2-[^18^F]FDG, 2-deoxy-2-[^18^F]fluoro-d-glucose.

Because of the recurrent FUO and the persistent elevated hsCRP levels, the patient received a full screening for potential viral infections (hepatitis-B-virus surface antigen and antibody, hepatitis-C-virus antibody, HIV-1/2 antigen/antibody, HIV-1/2, PCR acute influenza virus-A and influenza virus-B RNS antigens/antibodies, PCR acute respiratory syncytial viruses RNS as well PCR coronavirus SARS-CoV-2), which was negative. Urine test was negative. Blood cultures were taken and did not show bacterial growth in both aerobic and anaerobic cultures. Echocardiography and chest radiography were also performed. Despite antibiotic treatment, the patient still reported recurrent FUO and persistent elevated levels of hsCRP. Then, 2-[^18^F]FDG PET/CT at the Department of Nuclear Medicine at Vienna General Hospital was performed to find a possible focus of inflammation or infection. The PET only showed multiple 2-[^18^F]FDG hotspots in both thyroid lobes, mostly on the right (Figure 1).

**Figure 2:**
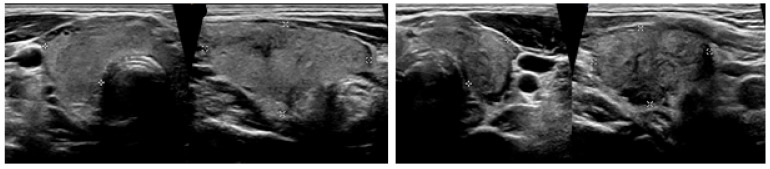
**Ultrasound images at the time of the diagnosis of SAT.** The total volume of the thyroid gland was about 18 mL (right lobe 10.1 mL and left lobe 7.2 mL) with heterogeneous echotexture and hypoechoic areas with ill-defined margins. SAT, subacute thyroiditis.

It was noted in the report that the finding could be compatible with thyroiditis and that the patient was promptly presented at our Out-Patients Department of Thyroid Diseases, where an ultrasound of the neck was performed. It showed an enlarged thyroid gland with heterogeneous echostructure. Both lobes had hypoechoic areas with ill-defined margins. There was one nodule seen in the right thyroid lobe, measuring up to 1 cm (Figure 2). Color flow Doppler showed reduced blood flow in both lobes.

Because of the recent contrast medium administration for the PET/CT imaging, [^99m^Tc]Tc-pertechnetate thyroid scintigraphy was not performed. The thyroid function test revealed primary hyperthyroidism with elevated fT) (11.10 pg/mL) and fT4 (4.36 ng/mL) and suppressive TSH (<0.01 mIU/L). Thyrotropin receptor antibody and thyroperoxidase antibody were negative. Thyroglobulin levels were elevated (420 ng/mL), and thyroglobulin antibody levels were within the normal reference range at 26 IU/mL. hsCRP was elevated to 3.66 mg/dL. Subacute thyroiditis was diagnosed. Other possible causes such as Grave’s disease or Hashimoto´s autoimmune thyroiditis or acute infectious thyroiditis were unlikely.

### 2.3. Treatment

Upon diagnosis, a treatment with prednisolone at a dose of 50 mg once a day was started for a week. A steroid-tapering regimen was undertaken for the patient with a reduction to prednisolone 25 mg once a day for a week, followed by reduction to 12.5 mg once a day for a week, with further reductions in the following weeks. This steroid-tapering regimen is performed to decrease the dosage of prednisone to the minimum required for symptomatic relief with periodic monitoring of thyroid function every three to four weeks. Furthermore, a therapy with a proton pump inhibitor was instilled during the period of oral steroid treatment.

### 2.4. Outcome and Follow Up

Three weeks later, the patient reported a drastic improvement of clinical symptoms, with no fever, no feeling of pressure on the neck, and no difficulty swallowing. The hsCRP levels had dropped to 0.12 mg/dL, and his thyroid function test showed normal results with fT3 at 3.12 pg/mL, fT4 at 0.97 ng/mL, and TSH at 2.03 mIU/L. The patient was recommended to continue with the steroid-tapering regimen. After 8 weeks, [^99m^Tc]Tc-pertechnetate thyroid scintigraphy was performed and showed a homogeneous [^99m^Tc]Tc-pertechnetate uptake in both thyroid lobes with slightly decreased diffuse tracer uptake in the left thyroid lobe. No evidence of potential pathological areas of increased or decreased uptake was found. The thyroid uptake of [^99m^Tc]Tc-pertechnetate at 20 min after the tracer injection was 1.1%. Furthermore, his thyroid function tests showed normal results with fT3 at 3.25 pg/mL, fT4 at 1.04 ng/mL, and TSH at 2.82 mIU/L. hsCRP levels were at 0.34 mg/dL. In view of complete resolution of his symptoms, the steroid therapy was stopped. Further follow up was scheduled.

## 3. Discussion

In this case report, we analyzed a case of atypical SAT originally presenting with a clinically history of FUO and elevated levels of hsCRP, without the typical symptom of neck pain. The patient underwent 2-[^18^F]FDG PET/CT examination, and we observed a diffuse, inhomogeneous 2-[^18^F]FDG uptake in the thyroid, which led us to examine the thyroid, resulting in a final diagnosis of SAT. Other causes of FUO or IUO were not found.

Thyroid gray-scale ultrasonography combined with color Doppler is a very effective, validated, and sensitive tool in diagnosing and monitoring thyroid disorders. Park et al. suggested that heterogeneous diffusely or focally ill-defined elongated hypoechoic areas without flow are typical findings of SAT [10]. Even if the inflammation process involves the entire thyroid, the patients with SAT could present an asymmetric enlargement of the gland. A further validated imaging modality in patients with thyroid diseases is the [^99m^Tc]Tc-pertechnetate scintigraphy. In the acute phase of SAT, a [^99m^Tc]Tc-pertechnetate scan shows markedly reduced uptake in the entire gland [11]. Thyroid gray-scale ultrasonography and [^99m^Tc]Tc-pertechnetate scintigraphy are generally performed promptly in patients with thyrotoxicosis and typical neck pain, because of suspected thyroiditis. Generally, both these imaging investigations in association with a thyrotoxicosis on the blood tests could allow a timely diagnosis of SAT, without resorting to 2-[^18^F]FDG PET/CT, a more expensive examination with a higher radiation burden. Nevertheless, our patients presented only a previous clinical history of FUO, without the typical clinical and biochemical features of a thyroiditis. Precisely, in patients with light or absent neck pain, with only elevated hsCRP on laboratory samples, and with a clinical history of FUO or IUO, 2-[^18^F]FDG PET/CT may be helpful for the differential diagnosis of SAT, according with our case and with those reported by Yoshida et al. and Lambert et al. [12,13].

Yen et al. described a case of localized SAT in a patient, which underwent 2-[^18^F]FDG PET/CT. A focal, intensely hypermetabolic thyroid lesion was reported, and the probability of malignancy was also considered [9]. Moreover, localized hypoechoic lesions on ultrasound and asymmetry of the thyroid are also common findings for a thyroid carcinoma. Nevertheless, the common forms of thyroid carcinomas (papillary and medullary thyroid carcinomas) are usually hypervascular. Zacharia et al. explained that, in this controversial case, a fine-needle aspiration biopsy is necessary to confirm the diagnosis [14].

This case illustrates the significant role of 2-[^18^F]FDG PET/CT as a diagnostic tool in patients with IUO or FUO and persistent elevated levels of hsCRP. FUO is defined as illness for at least 3 weeks, body temperature over 38.3 °C on several occasions, and no specific focus despite extended diagnostics [5]. The three major categories that account for most FUOs are infections, malignancies, and non-infectious inflammatory diseases. The diagnostic approach in FUO and IUO includes repeated physical and laboratory investigations, blood cultures, urine cultures, and standard imaging. Our case supports the significant diagnostic role and impact of 2-[^18^F]FDG PET in the diagnostic workup of patients with FUO or IUO [4,5]. In the present case of atypical SAT, a prior history of viral infection was unclear, and the typical symptom of neck pain was initially absent. Considering the reported aortic valve vitium, the first clinical suspicion was infective endocarditis. The laboratory tests as well the echocardiography excluded this as well other infections. Due to initial suspicion of acute sinusitis, an antibiotic treatment was undertaken. However, the levels of hsCRP remained elevated for some weeks, even after antibiotic therapy. The clinical manifestation suspected for SAT appeared only several days before performing the 2-[^18^F]FDG PET/CT scan.

2-[^18^F]FDG is an analog of glucose and is taken up by living cells via cell membrane glucose transporters and subsequently phosphorylated with hexokinase inside most cells. 2-[^18^F]FDG accumulation in tissue is proportional to the amount of glucose utilization, and it can be used to obtain quantitative parameters concerning the metabolic activity of target tissues [1,4]. It is known that 2-[^18^F]FDG PET is a well-established method in oncological imaging. However, it also has an important role in identifying infection and areas of inflammation [4]. To our knowledge, there have only been a few published cases of patients suffering from SAT who underwent 2-[^18^F]FDG PET imaging [7,8,9]. In two of these cases, the patient demonstrated thyrotoxicosis [7,8], and in one case report, the patient had normal thyroid function [9]. 2-[^18^F]FDG PET imaging showed in all three cases an asymmetrical increased tracer uptake in the thyroid [7,8,9]. Our patient presented with diffuse, inhomogeneous 2-[^18^F]FDG avidity in the entire thyroid, which is consistent with the previous reports [7,8,9].

## 4. Conclusions

The present case report may highlight the potential diagnostic value of 2-[^18^F]FDG PET/CT in case of atypical SAT, helping in the subsequent clinical and therapeutic patient management, and may have clinical implications in the differential diagnosis of FUO or IUO.

## Data Availability

Not applicable.

## Abbreviations

2-[18F]FDG	2-deoxy-2-[18F]fluoro-d-glucose
fT3	free triiodothyronine
fT4	free thyroxine
FUO	fever of unknown origin
hsCRP	high-sensitivity C-reactive protein
IUO	inflammation of unknown origin
PET/CT	positron emission tomography/computed tomography
SAT	subacute thyroiditis
TSH	thyroid-stimulating hormone
